# A Mobile Device App to Reduce Time to Drug Delivery and Medication Errors During Simulated Pediatric Cardiopulmonary Resuscitation: A Randomized Controlled Trial

**DOI:** 10.2196/jmir.7005

**Published:** 2017-02-01

**Authors:** Johan N Siebert, Frederic Ehrler, Christophe Combescure, Laurence Lacroix, Kevin Haddad, Oliver Sanchez, Alain Gervaix, Christian Lovis, Sergio Manzano

**Affiliations:** ^1^ Department of Pediatric Emergency Medicine Geneva Children's Hospital University Hospitals of Geneva Geneva Switzerland; ^2^ Department of Radiology and Medical Informatics Division of Medical Information Sciences University Hospitals of Geneva Geneva Switzerland; ^3^ Department of Health and Community Medicine Division of Clinical Epidemiology University of Geneva and University Hospitals of Geneva Geneva Switzerland; ^4^ Department of Pediatric Surgery Geneva Children’s Hospital University Hospitals of Geneva Geneva Switzerland

**Keywords:** resuscitation, medication errors, pharmaceutical preparations, pediatrics, biomedical technology

## Abstract

**Background:**

During pediatric cardiopulmonary resuscitation (CPR), vasoactive drug preparation for continuous infusion is both complex and time-consuming, placing children at higher risk than adults for medication errors. Following an evidence-based ergonomic-driven approach, we developed a mobile device app called Pediatric Accurate Medication in Emergency Situations (PedAMINES), intended to guide caregivers step-by-step from preparation to delivery of drugs requiring continuous infusion.

**Objective:**

The aim of our study was to determine whether the use of PedAMINES reduces drug preparation time (TDP) and time to delivery (TDD; primary outcome), as well as medication errors (secondary outcomes) when compared with conventional preparation methods.

**Methods:**

The study was a randomized controlled crossover trial with 2 parallel groups comparing PedAMINES with a conventional and internationally used drugs infusion rate table in the preparation of continuous drug infusion. We used a simulation-based pediatric CPR cardiac arrest scenario with a high-fidelity manikin in the shock room of a tertiary care pediatric emergency department. After epinephrine-induced return of spontaneous circulation, pediatric emergency nurses were first asked to prepare a continuous infusion of dopamine, using either PedAMINES (intervention group) or the infusion table (control group), and second, a continuous infusion of norepinephrine by crossing the procedure. The primary outcome was the elapsed time in seconds, in each allocation group, from the oral prescription by the physician to TDD by the nurse. TDD included TDP. The secondary outcome was the medication dosage error rate during the sequence from drug preparation to drug injection.

**Results:**

A total of 20 nurses were randomized into 2 groups. During the first study period, mean TDP while using PedAMINES and conventional preparation methods was 128.1 s (95% CI 102-154) and 308.1 s (95% CI 216-400), respectively (180 s reduction, *P*=.002). Mean TDD was 214 s (95% CI 171-256) and 391 s (95% CI 298-483), respectively (177.3 s reduction, *P*=.002). Medication errors were reduced from 70% to 0% (*P*<.001) by using PedAMINES when compared with conventional methods.

**Conclusions:**

In this simulation-based study, PedAMINES dramatically reduced TDP, to delivery and the rate of medication errors.

## Introduction

Immediate (level 1) triage represents 175,000 patient visits every year in US pediatric emergency departments (PED) [1]. Among them, 6700 to 15,000 cases are due to out-of-hospital cardiac arrest (OHCA) [2-4], including 6000 related to nontraumatic origins [5], and 5800 to 10,000 to in-hospital cardiac arrest (INHCA) [6,7]. In our institution in 2014, cardiopulmonary resuscitation (CPR) accounted for 0.5% of almost 28,000 pediatric visits (0-15 years old). In CPR, time is a decisive success criterion. During the first 15 min, survival and favorable neurological outcome decrease linearly by 2.1% and 1.2% per minute, respectively [8], and are negatively affected by drug preparation (TDP) and delivery time (TDD) [9]. In a study with adults in cardiac arrest, the chance of return of spontaneous circulation (ROSC) was decreased by 4% for every 1-min delay in delivery of vasopressor [10].

Prolonged resuscitation time may result from TDP [11]. During some critical situations such as postcardiac arrest ROSC or septic shock, preparing intravenous (IV) vasoactive drugs for continuous infusion is particularly challenging. Quickly, accurately, and safely preparing and administering drugs in a stressful environment is complex and time-consuming [12-14]. The need for individual specific weight-based drug dose calculation and preparation and a lower dosing-error tolerance [15] place children at higher risk than adults for errors [16-18] and may result in life-threatening outcomes. Medication errors have been reported in up to 41% of pediatric resuscitations, the most common being incorrect medication dosage found in up to 65% of cases [19]. Proper preparation and delivery of these drugs could favorably affect the pediatric CPR outcomes.

To address these problems, we followed a cognitive and evidence-based ergonomic driven approach [20] to develop an innovative and customizable tablet app, called Pediatric Accurate Medication in Emergency Situations (PedAMINES). This app was designed to support nurses and physicians step-by-step from order to delivery of a wide range of drugs in real time, including those requiring continuous infusion [21]. The development of the app was followed by a study aiming to assess its impact on the error rate and time needed from drug prescription to administration. We hypothesized that PedAMINES would first reduce the TDP and TDD, and second, reduce medication errors during pediatric CPRs when compared with conventional preparation methods.

## Methods

### Study Design

**Figure 1:**
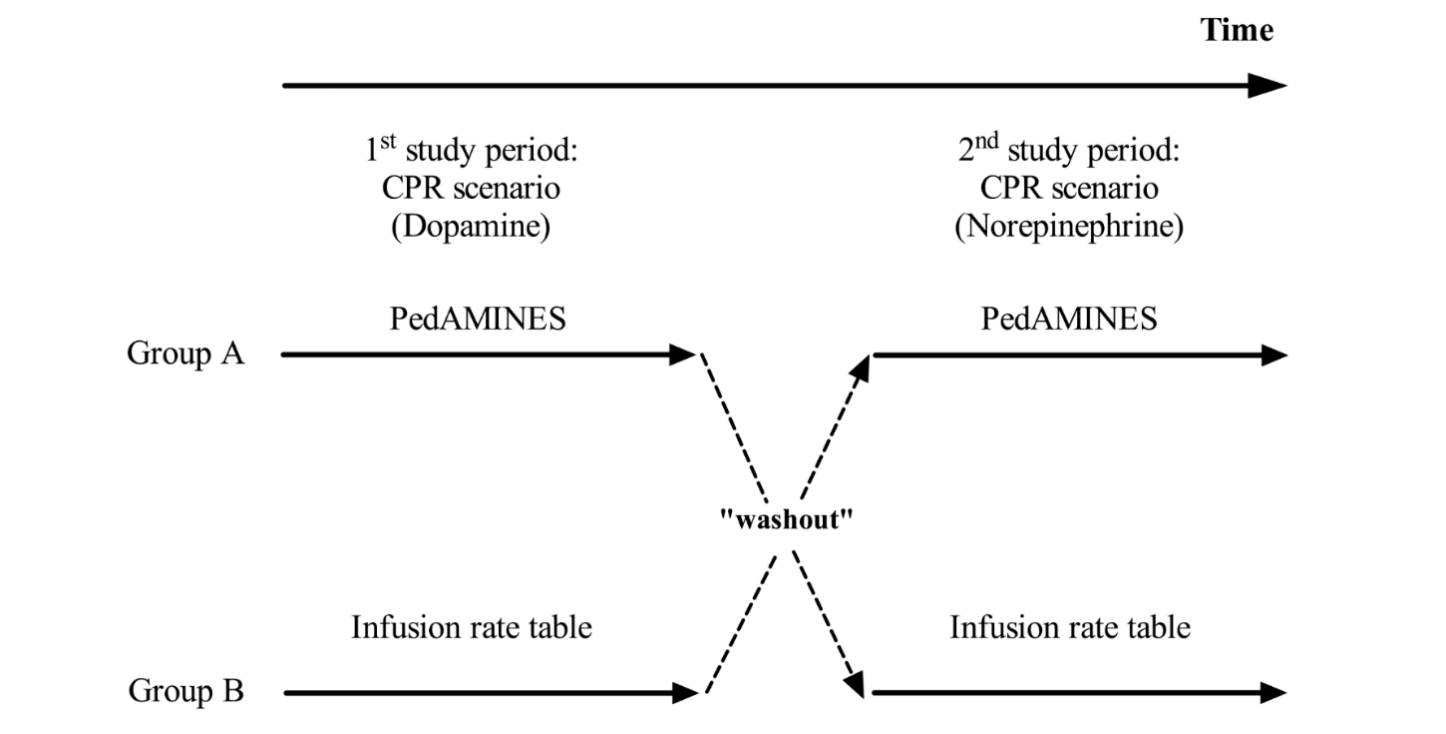
Study design: A two-period, randomized controlled, two-arm crossover study.

The study was a prospective, randomized controlled crossover trial with 2 parallel groups (Figure 1) comparing PedAMINES [21] with a conventional and internationally used drugs infusion rate table method (Frank Shann conventional drug infusion rate table [22]; Multimedia Appendix 1) in the preparation of continuous drug infusion, during a standardized simulation-based pediatric postcardiac arrest scenario. No changes were made on the app or on the intervention during the study.

### Selection of Participants

Certified pediatric emergency nurses were eligible if they were actively practicing in our PED. Shift-working nurses were randomly recruited on the day of the study by a blinded, noninvestigator, person on a random list. Written informed consent was obtained from all the participants before their voluntary involvement. Study participants were neither involved in the design of the app, nor in the study design, choice of outcome measures, or the study conduct. A senior specialized nurse in pediatric emergency medicine, simulation, and teaching, being an investigator of the study, has participated in the app and study design. No participants were asked to advice on interpretation or writing up of results. Results of the study were disseminated to the participants after the completion of the study.

### Setting

**Figure 2:**
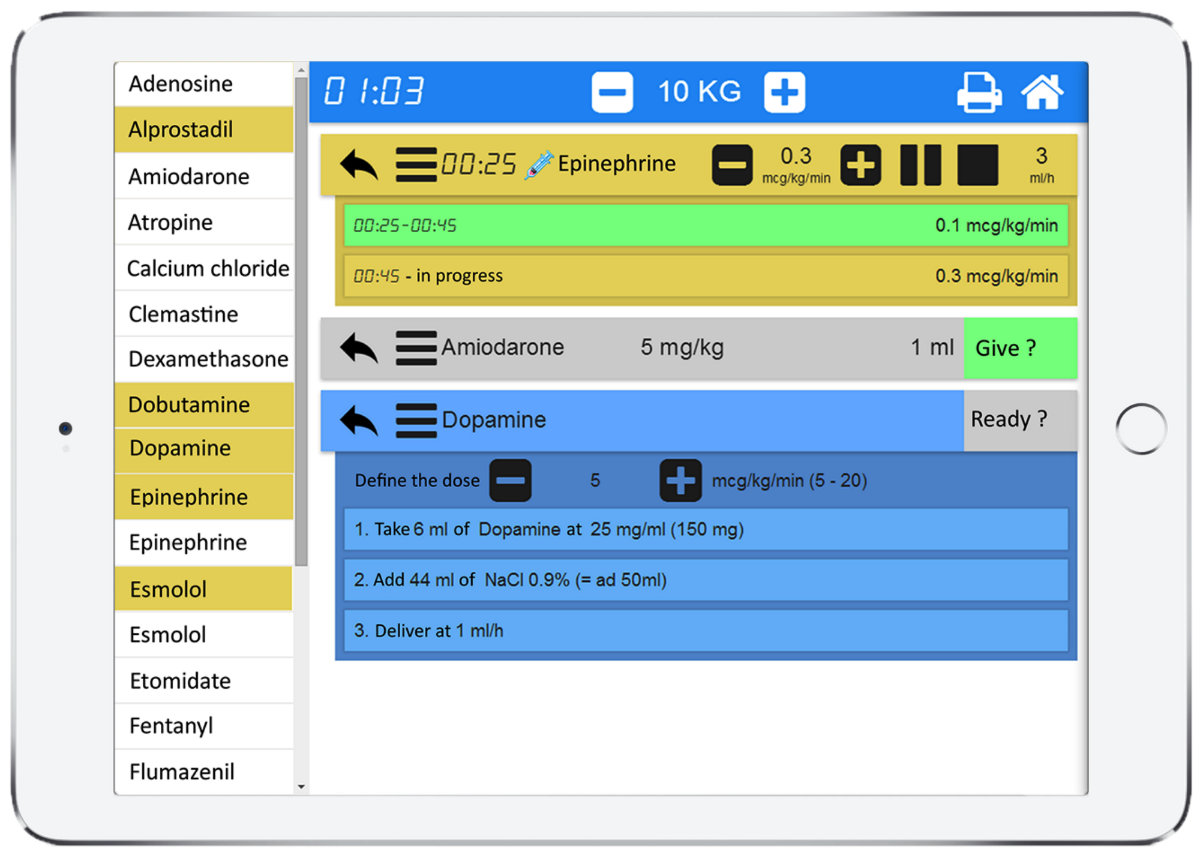
PedAMINES screenshot. List of bolus IV drugs (white boxes) and drugs for continuous infusion (yellow boxes) are selectable in the left margin of the application. The right window shows drugs selected by the nurse for a ten kilograms child. In this screenshot example, epinephrine is being delivered at an infusion rate of 0.3 mcg/kg/min. Amiodarone is selected and ready to be injected, waiting for nurse’s confirmation. Dopamine is being prepared by the nurse following a descriptive and detailed path automatically calculated by the application. The printer logo in the upper right corner indicates that all actions performed by the nurses are sequentially saved in historic files that can be retrieved and printed at any time.

The study was conducted in a PED of a tertiary hospital with approximately 28,000 visits per year. The PedAMINES app lists all the available resuscitation drugs, with doses automatically adapted to the weight or age of the patient. Evidence-based development of tools is an efficient way to develop apps that support clinicians [20]. The development of PedAMINES followed a user-centered approach with emergency department (ED) caregivers, as well as software developers and ergonomists. This team worked tightly together and the app development was mainly based on CPR observations and focus groups [21]. In this study, 6 drugs for continuous infusion and 19 drugs for direct IV injection were listed in the PedAMINES app and at the nurse’s disposal. The list can be expanded and customized according to users’ desires and to local drugs habits. By a simple touch, any of the listed drugs can be selected and its preparation detailed according to a standardized and simplified path. In the case of a continuous infusion, this path is composed of 3 steps: (1) drug selection, (2) dilution of the initial drug concentration, and (3) conversion of the prescribed dose rate in μg/kg/min into infusion pump rate in ml/h. For each drug, the exact amount to prepare is clearly displayed and thus avoids the necessity for calculations (Figure 2). This is based on the app’s ability to automatically calculate the optimal weight-based final infusion pump rate and to describe the preparation sequence required to achieve it, independently of the nurse competency in this domain. The nurse may, at any time, interact with the app. The user can start, pause, stop, increase, or diminish the perfusion rate. Multiple drugs can be prepared and run in parallel. All actions performed by the nurses were sequentially saved locally on the device in historic files to preserve information that can be retrieved at any time for debriefing or medicolegal purposes. Historic files can also be erased or safely exported and saved on the institutional electronic health record.

### Intervention and Resuscitation Scenario

We created a standardized simulation scenario on a high-fidelity manikin (Laerdal SimBaby) in the pediatric shock room of our PED. Consistent with standard emergency medicine practice, we created resuscitation teams comprising the study-participating nurse, an emergency pediatrician leading the resuscitation and being a Pediatric Advanced Life Support (PALS) instructor, and a second nurse (both part of the investigators team) to assist with resuscitation by performing chest compressions and bag-valve-mask ventilation according to the pediatrician instructions. A certified technician operated the simulator. Except for the participating nurse, the members of the resuscitation team were unchanged across all the scenarios and were the investigators of the study.

On the day of participation, nurses were given a survey collecting data regarding their demographics, nursing, and computer experience. After random allocation, each participating nurse received a standardized 5-min training session on how to use the app PedAMINES to familiarize them uniformly with it. Then, the nurses were asked to perform a 15-min highly realistic CPR scenario, including postreturn of spontaneous circulation (ROSC). It was standardized to follow the same chronological progression and provided on the same manikin. The scenario was conducted in situ in our shock room to increase realism. When entering the shock room, the nurses were asked to assist the physician to perform a 2-min full course massage and ventilation (15:2 ratio) maneuver for a child with asystole to increase their own stress level. On the basis of the American Heart Association (AHA), pediatric cardiac arrest algorithm for asystole, a bolus of 0.01 mg/kg epinephrine (0.1 mL/kg of 1:10,000 concentration) was administered. ROSC ensued with hypotension. At that time, a clinical statement to recognize the life-threatening condition of the patient, including his weight and age, was given to the nurses. The nurses were then asked to prepare a 5 μg/kg/min continuous infusion of dopamine for a 7-kg boy either with the help of PedAMINES first (allocation group A) or following the conventional method first (ie, Shann infusion rate table [22], group B; Multimedia Appendix 1). All participants had equal experience and competence with the Shann method. At the end of the instruction, the timed scenario began. To ensure that participants heard and understood the order correctly, they had to confirm it verbally, and written transcriptions were checked and video-recorded. When the drug was ready to be injected, the nurse was asked to deliver it to the patient using a syringe pump already in place. The nurse was then asked to perform a 1-min “washout” distraction maneuver by aspirating secretions in the throat of the manikin. At this moment, the crossover occurred. The nurse was asked to prepare a 0.1 μg/kg/min continuous infusion of norepinephrine by crossing the procedure (ie, group A allocation having used PedAMINES before was asked to use the conventional method, whereas group B allocation having used the conventional method before was asked to use PedAMINES; Figure 1). At the end of the order, the nurse was asked to prepare the drug. Given that norepinephrine preparation could present a challenge by requiring a more complex decimal-point dependent calculation with the Shann method, we rendered the tasks uniform by ordering a decimal-point final volume calculation for both Frank Shann and PedAMINES preparation methods. When the drug was ready to be injected, the nurse was asked to deliver it to the patient using a second syringe pump already in place. The delivery of both drugs required programming the same pump in a similar manner among all participants. Time elapsed after drug preparation until its delivery, that is, time needed to set up the pump, was assessed for all participants to ensure uniformity among participants. The measured deviation between the amount of drug delivered and the actual prescribed dose was given by the amount of drug in the syringe. This was verified by an examiner during the scenario and video-recorded. The beginning of the injection corresponded to the end of the scenario. At that time, the nurse had to recall and describe precisely how she prepared both the drugs.

### Outcome Measures

The primary outcome was the elapsed time in seconds, in each allocation group, between the oral prescription by the physician and TDD by the nurse. TDD included TDP completion by the nurse. The secondary outcome was the medication dosage error rate in each allocation group, during the sequence from drug preparation to drug injection. Regarding both outcomes, we considered a 15 s difference in delivery of resuscitation drugs [23], and a 30% difference in the rate of medication errors to be clinically significant and sufficient to modify practice. At the end of the scenario, a 2 question questionnaire using a 10-point Likert scale (scored from 1 to 10 to avoid neutral answers) was submitted to participants. The questionnaire measured (1) the overall stress perceived (the question was “On a scale of 1 to 10, how much stress did you feel during the whole resuscitation scenario?”) and (2) the satisfaction about the preparation method used during the resuscitation scenario (the question was “On a scale of 1 to 10, how much satisfaction did you get during the resuscitation scenario with the help of PedAMINES, and with the help of the infusion rate table?”).

### Methods of Measurement and Data Collection

Data collected during the scenario included (1) TDP, (2) TDD, and (3) final delivered drug concentration in μg/ml and infusion rate in μg/kg/min. All the actions (ie, primary and secondary outcomes) performed by the nurses during the scenario were automatically recorded and stored by the responsive simulator detectors and by several video cameras. To avoid assessment bias, 2 evaluators then independently reviewed these video recordings. In case of disagreement, a third independent evaluator helped reach a consensus. All actions performed with PedAMINES were automatically saved locally in log files for further analysis. Data were manually retrieved and entered into a Microsoft Excel spreadsheet (version 2011). The statistical software GraphPad Prism version 6.0h (GraphPad Software, Inc) was used for graph figures. Stata/IC version 14 (StataCorp) was used for descriptive analyses, and R version 2.15.2 (R Foundation for statistical computing) was used for statistical tests and 95% CI.

### Sample Size

The primary objective of this study was to detect a difference in TDD of vasoactive drugs between the 2 groups. The sample size was calculated to detect a 15 s decrease in TDD between 2 independent groups with a power of 90% and a 2-sided risk alpha of .05. A previous study with pediatric emergency nurses has shown a median TDD of 69 s for the first dose of vasoactive drug to be given as direct IV infusion [23]. Assuming a standard deviation (SD) of 9 s for TDD in each group (based on a similar SD of 10 s estimated by Moreira et al [23]), 9 participants per group were required. To prevent a potential loss of power due to misspecification of assumptions, it was necessary to recruit 10 nurses per group (total sample size: 20 nurses). In case of a carryover effect, this sample size calculation was sufficient to evaluate PedAMINES’s effect within the first period of the trial.

### Randomization and Blinding

We randomly assigned nurses in a 1:1 ratio with a Web-based software [24]. Blinding to the purpose of the study during recruitment was maintained to minimize preparation bias. Nurses were unblinded after randomization. Allocation concealment was ensured with sealed envelopes and was not released until the nurses started the scenario.

### Statistical Analysis

#### Primary Outcome

For TDD (and TDP), the mean times were reported with 95% CI for each arm and each study period to investigate a potential carryover effect. As a carryover effect was suspected, intervention arms were compared within each study period using *t* tests for independent groups. No paired data were compared.

#### Secondary Outcomes

The rate of medication errors was the proportion of nurses making a preparation error. The rate of medication errors was reported with 95% CI (Clopper-Pearson method) with each method and by study period to investigate a potential carryover effect. The error rates for each method were globally assessed and compared using McNemar test as observations were paired. Differences in error rates were reported with 95% CIs.

Errors were also measured as the deviation in percent from the amount of delivered drug compared with the original dose prescribed by the physician. Absolute deviation was analyzed. The mean (SD) difference in deviation obtained with each method was reported with 95% CI. A *t* test for paired data was used to compare interventions. Mean differences were also reported by randomized group and by crossover period. Means and SDs were determined for stress and satisfaction scores of individuals for each questionnaire item and reported with descriptive statistics.

### Ethics and Informed Consent

The study was approved by the institutional ethics committee and a trial registration number was not required. If we regard simulation as a translational science with outcomes categorized as T1 (results achieved in the simulated setting), T2 (improved health care delivery in the real clinical setting), and T3 (improved patient outcomes), our trial was a T-1 level study and, as for many other simulation studies, did not require registration according to ICMJE definition. Written informed consent was obtained from all participants before their voluntary involvement. It was conducted in accordance with the principles of the Declaration of Helsinki, the standards of Good Clinical Practice, and Swiss regulatory requirements.

## Results

### Study Participants

**Figure 3:**
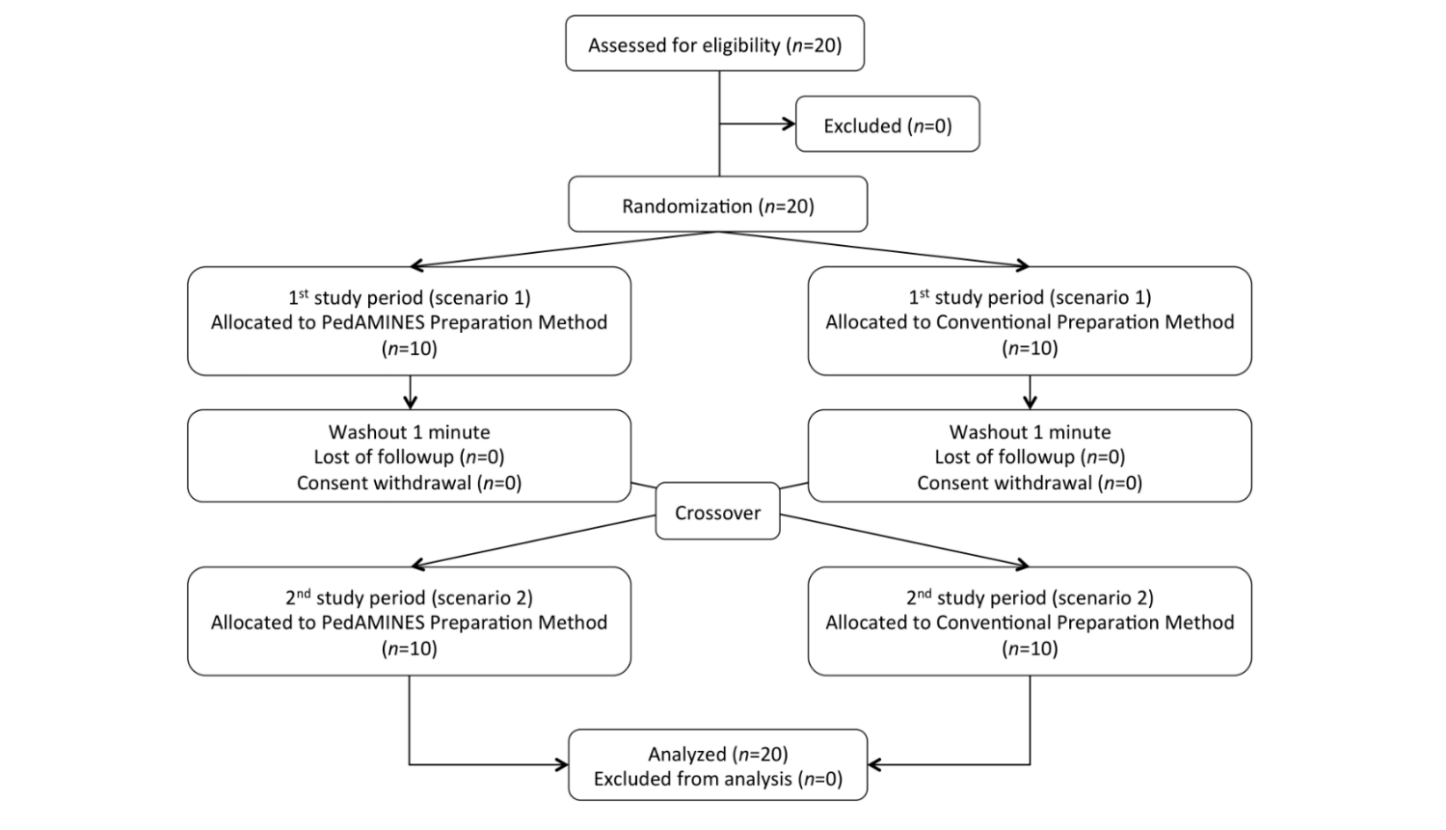
Patient flowchart for randomized controlled trial on preparation of continuous drug infusion by nurses in simulation-based pediatric cardio-pulmonary resuscitation cardiac arrest scenario.

In June 2015, 20 certified pediatric emergency nurses participated and completed the study with no dropout (Figure 3). The demographic results are summarized in Table 1.

**Table 1:** Participants’ demographic and clinical characteristics.

Demographics and clinical characteristics	Randomization arm (first study period)	
	PedAMINES^a^ (n=10)	Infusion rate table (n=10)
Age (years), mean (SD)	42.9 (6.4)	42.4 (10.5)
Sex (female), n (%)	8 (80)	7 (70)
Number of years since nurse certification, mean (SD)	19.2 (7.7)	18.5 (11.7)
Number of years since pediatric ED^b^ certification, mean (SD)	9.9 (6.4)	9.8 (6.5)
Own and use a tab or mobile phone at home, n (%)	9 (90)	9 (90)
CPRs^c^ having required vasoactive drugs Preparation for continuous infusion in the past 3 years, median (interquartile range)	2.0 (0.0-2.30)	1.0 (1.0-2.0)
Simulated CPR scenarios in the past 3 years, median (interquartile range)	7.0 (3.8-12.0)	5.5 (3.8-8.3)

^a^PedAMINES: Pediatric Accurate Medication in Emergency Situations.

^b^ED: emergency department.

^c^CPR: cardiopulmonary resuscitation.

### TDP and TDD

**Figure 4:**
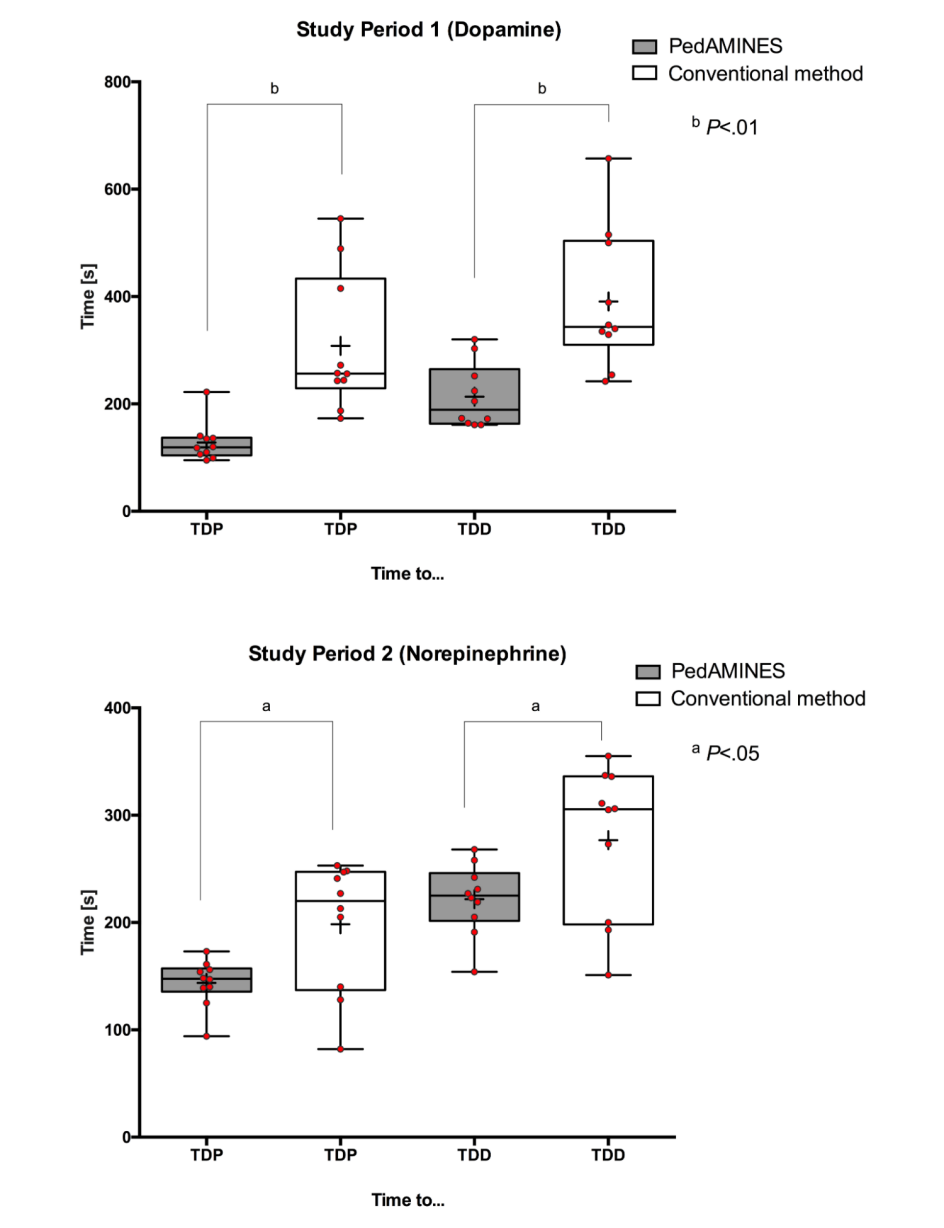
Study period 1 (Dopamine) and 2 (Norepinephrine). Boxplots of elapsed time to drug preparation (TDP) and to drug delivery (TDD) in intervention group (PedAMINES) and control group (conventional method). Solid horizontal lines denote median and interquartile ranges; the whiskers go down to the smallest value and up to the largest; + denotes mean. Red open circles denote each individual value. Time is expressed in seconds.

When using PedAMINES, the mean time either from drug prescription to preparation or to delivery was nearly equivalent in both study periods (128.1 s vs 143.7 s or 213.5 s vs 221.8 s, *P*=.71; Table 2). Using the conventional method, the mean time from prescription to preparation or to delivery was lower in the second period than in the first period (308.1 s vs 198.4 s, *P*=.03 and 390.8 s vs 276.7 s, *P*=.03, respectively; Table 2). These findings raised the suspicion of a carryover effect and comparisons between interventions were conducted for each period separately. In the first study period, the TDP was decreased by 180.0 s (95% CI 86.5-273.5, *P*=.002) with PedAMINES and TDD by 177.3 s (95% CI 79.7-274.9, *P*=.002; Figure 4). These gains in time represented 58% and 45% of the mean time, respectively, when using the conventional methods. The variability of individual recorded times was lower with PedAMINES (TDP upper range value=222 s, TDD=320 s) than with the conventional method (TDP upper range value=545 s [8 out of 10 measures were higher than 222], TDD=657 s; Figure 4). In the second study period, the TDP was decreased by 54.7 s (95% CI 10.3-99.1, *P*=.02) with PedAMINES and TDD by 54.9 s (95% CI 1.6-108.2, *P*=.04; Figure 4).

**Table 2:** Mean time in seconds to drugs preparation and delivery.

Mean time	First study period (Dopamine)		Second study period (Norepinephrine)	
	PedAMINES^a^ mean (95% CI)	Conventional method mean (95% CI)	PedAMINES mean (95% CI)	Conventional method mean (95% CI)
TDP^b^	128.1 (102.0-154.2)	308.1 (216.3-399.9)	143.7 (128.1-159.3)	198.4 (155.3-241.5)
SD	36.5	128.3	21.9	60.2
Time difference^c^	180.0 (86.5-273.5)		54.7 (10.3-99.1)	
TDD^d^	213.5 (170.6-256.4)	390.8 (298.3-483.3)	221.8 (198.2-245.4)	276.7 (226.2-327.2)
SD	59.9	129.3	33.0	70.6
Time difference^c,e^	177.3 (79.7-274.9)		54.9 (1.6-108.2)	

^a^PedAMINES: Pediatric Accurate Medication in Emergency Situations.

^b^TDP: time to drug preparation.

^c^Time difference represents time with the conventional method minus time with PedAMINES.

^d^TDD: time to drug delivery.

^e^See Multimedia Appendix 2 for TDP and TDD details for each nurse and for each drug, by study period.

### Medication Error Rate

As data did not support a carryover effect in the medication error rate, both the study periods were pooled. Of the 20 drug doses delivered with PedAMINES, none (0%) was associated with medication errors. Of the 20 drug doses prepared with conventional methods, 14 were incorrect (70%; 95% CI 45.7-88.1, *P*<.001). Among the 8 errors committed during the first study period, 5 (63%, 5/8) were critical overdose errors ranging from 100% to 5233% (mean 1864%) of the normal prescribed dose (Table 3). Among the 6 errors committed during the second study period, 3 (50%, 3/6) ranged from 19% to 138% (mean 59%) of the normal prescribed dose and 2 (33%, 2/6) miscalculated preparations reached the right final dosage by chance (Table 3).

**Table 3:** Drug doses errors and deviation from the prescribed doses.

Outcomes	First study period (Dopamine)		Second study period (Norepinephrine)^a^	
	PedAMINES^b^ n (%), 95% CI	Conventional method n (%), 95% CI	Conventional method n (%), 95% CI	PedAMINES n (%), 95% CI
All medication errors^c^ (n=10)	0 (0), 0-31	8 (80), 44-98	6 (60), 26-88	0 (0), 0-31
Unpaired medication errors difference (n=10)	8 (80), 41.4-97.5		6 (60), 21.3-88.5	
Paired medication errors difference (N=20)	14 (70), 42.2-88.1			

^a^See Multimedia Appendix 3 for drug doses errors details for each nurse and for each drug by study period.

^b^PedAMINES: Pediatric Accurate Medication in Emergency Situations.

^c^Proportion of dosage with an error, where n denotes the number of drug dose errors that actually occurred and N denotes the total number of opportunities for errors to occur.

### Questionnaire About Perceived Stress and Satisfaction

The questionnaire was completed and returned by 100% of the participants. Participants rated the overall perceived stress to be 7.1 (95% CI 6.1-8.1) on the Likert scale. They reported higher satisfaction when using PedAMINES for the preparation of drugs rather than conventional methods (9.3 [SD 1.2] vs 3.6 [SD 2.1], *P*<.001).

## Discussion

### Principal Findings

To our knowledge, this is the first study to investigate the benefit of a mobile app to improve delivery of continuous drug infusion during pediatric CPR. We found that TDP and TDD of vasoactive drugs for continuous infusion, as well as medication errors were dramatically reduced with the use of PedAMINES.

CPR is a continuum from the onset of resuscitation to immediate post-resuscitation care. Survival rate for CPR lasting less than 15 min is estimated at 41%, decreasing to 12% after 35 min [8]. A recent study reported that average time spent in the pediatric shock room was 46 min [25]. Care for postcardiac arrest patients is also time-sensitive. Postcardiac arrest syndrome (PCAS), characterized by brain injury, myocardial dysfunction, systemic ischemia or reperfusion response, and persistent precipitating disease, can develop precociously with poor outcome after ROSC [26,27]. Post-ROSC in-hospital mortality rates in children after nontraumatic OHCA or INHCA were estimated to be 70% [28] and 55% [29], respectively. If ROSC is quickly achieved and maintained after the onset of cardiac arrest, PCAS might be prevented [30]. Thus, early hemodynamic optimization improves the outcome of these patients [31,32]. The 2015 AHA guidelines recommended starting IV fluids and vasoactive drugs early in the postarrest phase, targeting a systolic blood pressure above the fifth percentile for age [33]. Commonly employed drugs for continuous infusion include dopamine (5-20 mcg/kg/min), norepinephrine, and epinephrine (both at 0.01-1 mcg/kg/min). These drugs should be available quickly. Moreira et al reported that the use of prefilled color-coded syringes during pediatric cardiac arrest can reduce the TDD of IV drugs by 27 s [23]. However, these results were limited to direct IV pushes requiring no prior preparation. Our work demonstrates that it is possible to drastically decrease TDD for IV drugs requiring complex upstream preparations and continuous infusion during the immediate postarrest phase. In our study, mean TDP, as well as mean TDD were significantly reduced by almost 180 s with PedAMINES. Even when considering the lower margins of errors of the CI, PedAMINES was still able to reduce TDP and TDD by approximately 1.5 min (4.5 min at the upper margins) and was largely higher than the minimal difference we set out to find when establishing the sample size. The interindividual upper time ranges were reduced by using the app, suggesting a worthwhile benefit if used in smaller hospitals where nurses and physicians are either rarely or not exposed to CPR, but have to use resuscitative drugs before patient transfer to a tertiary care center.

Although TDP and TDD were similar in the primary and secondary study periods when using PedAMINES, the gain provided by PedAMINES was less pronounced in the second study period, because TDP and TDD with the conventional method were lower among nurses with a PedAMINES experience than among nurses who had never used PedAMINES. The 1-min washout period may not have been long enough. Initially, we sought to estimate the effect of PedAMINES in the first study period. We wanted to determine whether changing habits with the use of our app by naïve nurses hitherto accustomed to a conventional preparation method resulted in a significant gain of time on the preparation of drugs compared with nurses using the conventional method. The second study period was intended to increase the overall power of our study. However, in this second period, the nurses could not be considered in exactly the same way as in the first period. Indeed, in the second period, we compared nurses who returned with a conventional preparation method after having used PedAMINES versus naïve nurses hitherto accustomed to a conventional preparation method that switched for PedAMINES for the first time. In any case, the intervention had a large impact in both periods. Given the carryover effect, the first study period was chosen as the most representative for the influence of PedAMINES on the primary outcome.

Medication errors are common in pediatric patients, accounting for 5-27% of all pediatric prescriptions and causing significant mortality and morbidity, including 7000 patient deaths each year in the United States [34]. One in every 32 prescriptions in a PED contains a 10-fold error on the recommended dose [17]. Kozer et al showed an error rate of 17% at the prescription level during pediatric resuscitations, with up to 10 times the recommended pediatric dose in more than 3% of these cases [35]. In the same study, 16% of the analyzed syringes showed a 20% dose deviation from the prescribed dose and up to 7% showed a deviation of more than 50%. Medication errors with infusions frequently result from mistake during preparation, due to wrong drug-volume calculations, imprecision with volume measurements or incorrect mixing during dilution [11,13,36]. Available conversion methods, such as infusion rate tables or nomograms [37], designed to simplify infusion rate calculation still remain difficult to use and subject to medication errors. Even small errors either in drug calculation or infusion pump flow rate may have a large detrimental impact on the amount of drug delivered [38-40]. This can be harmful for critical patients and even prove fatal [17]. In a study reporting 41% medication errors in pediatric CPRs, the most common error was drug dosage (65% of cases) [19]. Similarly, we found that using the conventional preparation method resulted in dosing errors in 70% of the preparations. Among them, 30% deviated from the original prescribed dose from 100% up to 5233% (ie, 2-fold to 53-fold overdose errors). Disruptive anxiety and exogenous conditions encountered during resuscitation increase the nurse’s cognitive workload and the risk of errors. The cognitive workload has also been shown to be higher when the task is less familiar, as typically seen during CPR [41], which remains uncommon in PED. For instance, at our PED, level one triage status patients represent 0.5% of the ED visits, a proportion similar to the 0.7% (+/- 0.2) seen in the US PED [1]. The lack of practice due to the rareness of certain pathologies or inexperience among the staff regarding vasoactive drugs preparation may add to the complexity of this phenomenon. Some authors have advocated replacing tasks requiring cognitive load during CPR by automated actions [23,41] as much as possible. By guaranteeing an automated, fast, and reliable conversion and preparation, medication errors were totally avoided using PedAMINES. It should be noted that we observed fewer medication errors in the control arm during the second study period, with only a single critical error of more than 20%. This was may be due to the higher number of vials required to prepare norepinephrine (3 vials) compared with that for dopamine (1 vial) or that nurses were a bit more trained (having just done the first scenario). While they had no impact on TDP and TDD, these additional steps of preparation have perhaps limited the rate of errors by strengthening the controls at each preparation step. As tasks were uniform, the complexity of dopamine and norepinephrine preparation by using either PedAMINES or Frank Shann method was similar and not expected to be responsible for the different rates of errors.

### Limitations

This study has some limitations. First, it was conducted during a resuscitation simulation-based scenario. This choice was related to the ethical and organizational difficulties of conducting studies with patients in critical situations. However, several studies have demonstrated the benefit of simulation as an investigative research methodology to answer research questions that otherwise could not be answered during CPR [42]. Interindividual diversity among patients and their diseases make CPR studies hard to standardize. Simulation-based CPR scenarios may overcome these limitations by providing a standardized and controlled environment, detailed feedback analysis of the resuscitation stages using audiovisual recordings, and reproducibility. Therefore, high-fidelity simulation has become essential to study resuscitations skills or technologies. We acknowledge that a simulation cannot reveal whether the intervention improves clinical outcome. However, till date, none of the results obtained from simulation-based CPR studies disagreed with those obtained from studies in real life, confirming our study design choice. Although the survival rate has complex and numerous components, it would be interesting to determine in further studies whether saving time and decreasing medication errors owing to the use of PedAMINES would translate into increase in patient survival in real life.

Second, the lack of immersive realism provided during simulated scenarios might directly affect the assessed outcomes. In this study, we used a high-fidelity manikin simulator, which is currently and widely recognized as providing the most realistic and high-yield immersive environment achievable in emergency training [43]. Realism in our study was reflected by the stress levels experienced by the participants. They quoted the simulation as highly stressful when compared with real CPR situations.

Third, accuracy of intervention delivery times could also limit some CPR studies when assessing “times to” outcomes. Conventional paper-based documentation practices used during resuscitation—simulated or not—are often unreliable and inaccurate, either by misreporting intervention delivery times or by missing the delivery completely [44]. In this study, all drug preparation times were chronologically saved in historic log files and videorecorded, rendering our outcomes highly accurate.

Fourth, many different methods are used in PEDs to prepare and administer infusion drugs such as the Frank Shann method or Broselow pediatric resuscitation medication or infusion guide. They all commonly require the user to perform some kind of calculation that may lead to errors and a longer TDP. As described by many authors, the Broselow pediatric emergency tape and affiliate pediatric resuscitation medication or infusion guide have been used with mixed results in many countries and are somehow inaccurate to predict actual weight in almost 20-30% of children, especially for under- and overweight children [45-52]. This may lead to medical errors due to incorrect dosing selection. In 2007, the American Academy of Pediatrics committee on Pediatric Emergency Medicine acknowledged, “although helpful, the Broselow tape is not ideal” [53]. Frank Shann method offers the possibility to precisely adjust the drug doses on patient’s actual weights and is used in many hospitals worldwide. This was the rationale to use it as a comparator in our study.

Finally, our study was not intended to compare PedAMINES with “smart” IV pumps. Standardizing drug concentrations of premixed drug and varying infusion rates with smart pumps implicate to deal with poor dose and rate precision in already unstable and critically ill children. As recently reported, no conclusive evidence shows that smart pumps do prevent medication errors and adverse drug events [54,55]. In addition, little is known about the kind of errors that still occur with their use. Moreover, the number of premixed drugs required, their chemical stability over time [56-59] and the lack of specialized pharmacy facility in many smaller hospitals or in other countries around the world, limit their use. Further studies comparing PedAMINES and smart pumps would be valuable.

### Conclusions

In summary, compared with a conventional and internationally used preparation method, we found that a mobile app developed following an evidence-based ergonomic-driven approach dramatically reduced TDP and TDD, as well as the medication error rate. The interindividual variance was also reduced by using the app, suggesting a worthwhile benefit to its use by nurses with different experience levels. A large multicenter randomized trial is further needed to assess this assumption in primary and secondary care hospitals.

## Appendix

Multimedia Appendix 1 Frank Shann Drug Doses.

Multimedia Appendix 2 The table details the time expressed in seconds to drug preparation (TDP) and delivery (TDD) for each nurse and for each drug, by study period.

Multimedia Appendix 3 The table details the deviation expressed in percent in drug dosage (overdose) from the prescribed dose for each nurse and for each drug, by study period.

Multimedia Appendix 4 CONSORT-EHEALTH checklist V1.6.1 [60].

## Abbreviations

CPR: cardiopulmonary resuscitation
ED: emergency department
PED: pediatric emergency department
PedAMINES: Pediatric Accurate Medication in Emergency Situations
TDD: time to drug delivery
TDP: time to drug preparation
