# Supplementary figures and images for: A Mobile Device App to Reduce Time to Drug Delivery and Medication Errors During Simulated Pediatric Cardiopulmonary Resuscitation: A Randomized Controlled Trial
Source: J Med Internet Res. 2017 Feb 1;19(2):e31. doi: 10.2196/jmir.7005 (PMC5311423; doi:10.2196/jmir.7005)

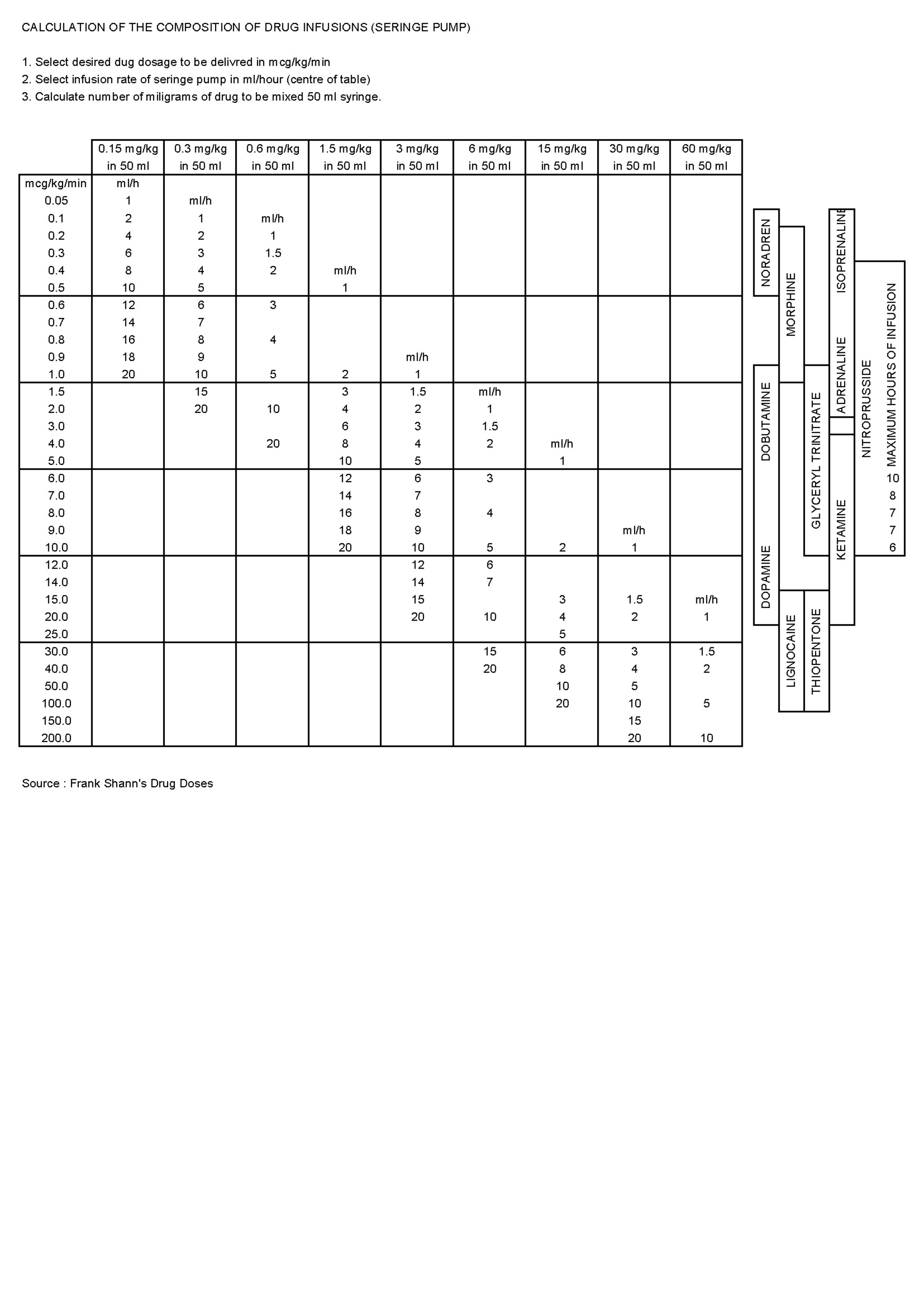

Supplement: Multimedia Appendix 1 [file jmir_v19i2e31_app1.png]

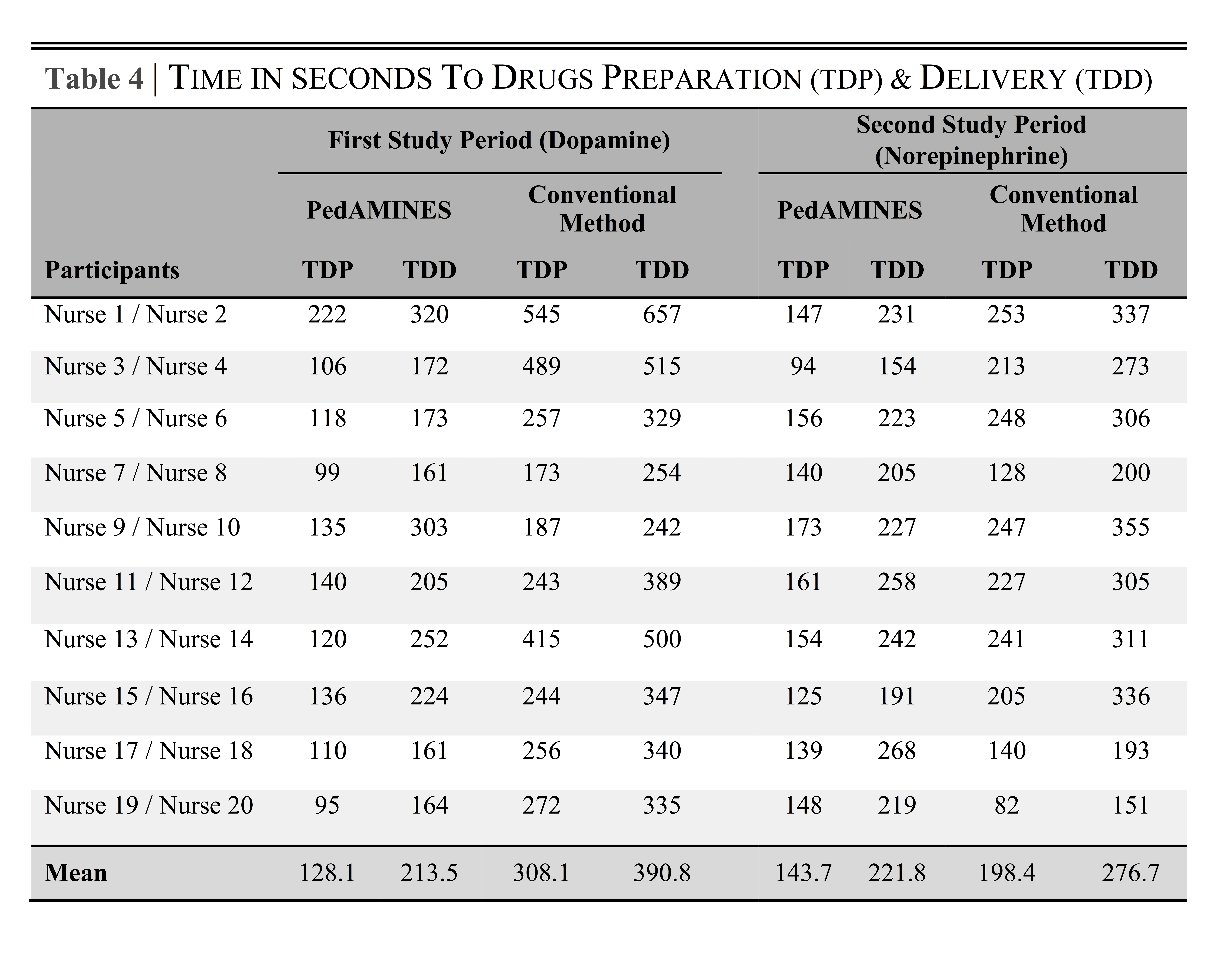

Supplement: Multimedia Appendix 2 [file jmir_v19i2e31_app2.png]

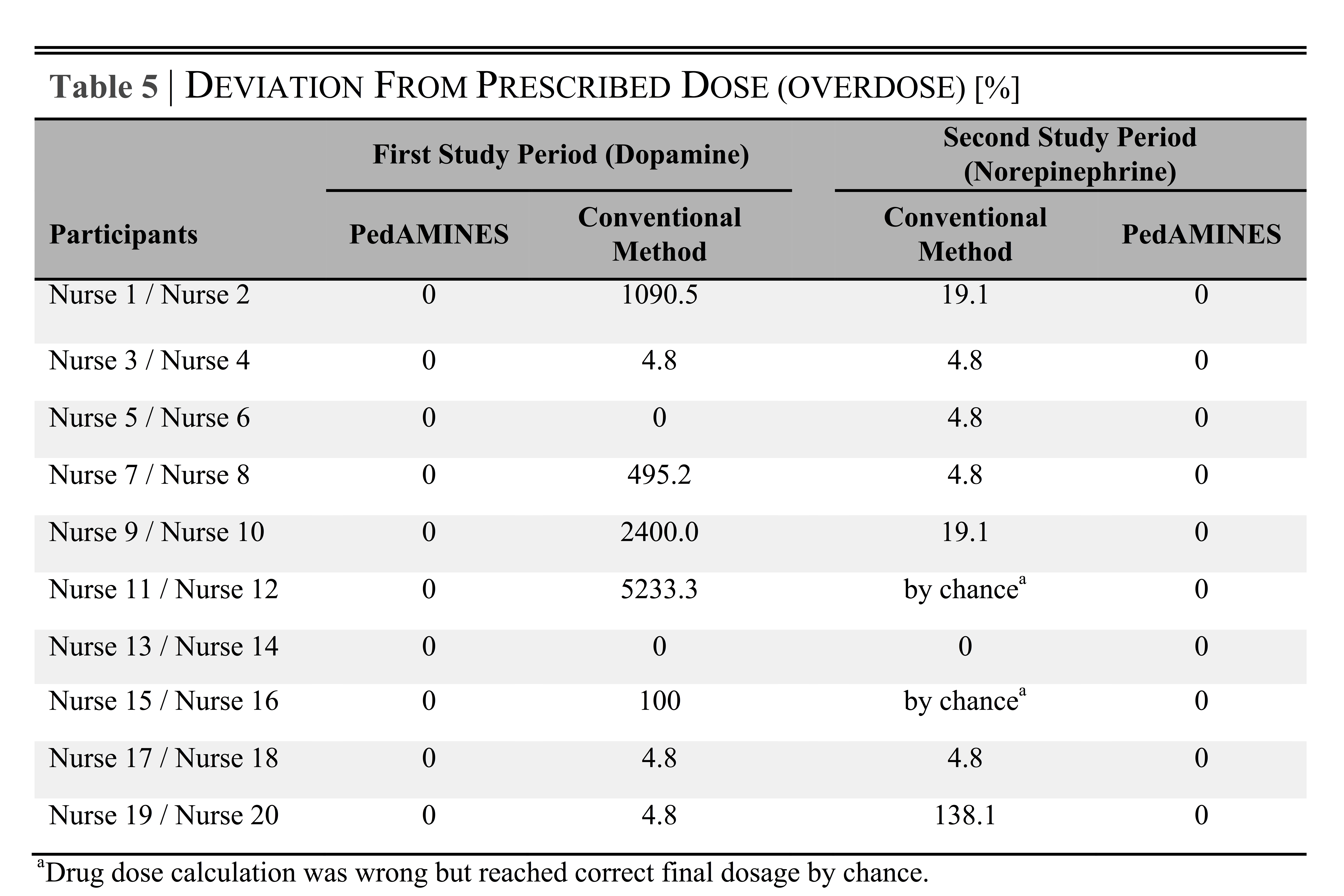

Supplement: Multimedia Appendix 3 [file jmir_v19i2e31_app3.png]
